# Minimally Invasive Cervical Styloidectomy in Stylohyoid Syndrome (Eagle Syndrome)

**DOI:** 10.3390/jcm12216763

**Published:** 2023-10-26

**Authors:** Jakub Bargiel, Michał Gontarz, Tomasz Marecik, Paweł Szczurowski, Krzysztof Gąsiorowski, Jan Zapała, Grażyna Wyszyńska-Pawelec

**Affiliations:** Department of Cranio-Maxillofacial Surgery, Jagiellonian University Medical College, 30-688 Cracow, Poland; michal.gontarz@uj.edu.pl (M.G.); tomasz.marecik@uj.edu.pl (T.M.); pawel.szczurowski@uj.edu.pl (P.S.); krzysztof.gasiorowski@uj.edu.pl (K.G.); jan.zapala@uj.edu.pl (J.Z.); grazyna.wyszynska-pawelec@uj.edu.pl (G.W.-P.)

**Keywords:** Eagle Syndrome, stylalgia, minimally invasive styloidectomy, MICS, styloidectomy, carotid artery syndrome, stylohyoid chain, styloid process

## Abstract

(1) Background: Stylohyoid syndrome, known as classical Eagle Syndrome (cES), is characterized by calcification of the stylohyoid chain with numerous nonspecific symptoms, mainly pain. This study introduces minimally invasive cervical styloidectomy (MICS). (2) Methods: MICS was performed on sixty-five patients diagnosed with classical Eagle Syndrome. Patients underwent meticulous differential diagnosis. Surgical plans were established based on the findings from neck angioCT. (3) Results: The healing process was uneventful, without significant complications. The overall success rate was 97.0%, with a follow-up of a minimum of six months. In one case, the surgery did not yield the desired improvement. In one case, a partial relapse of symptoms was observed. (4) Conclusions: MICS is a straightforward and efficient surgical treatment technique for stylohyoid syndrome.

## 1. Introduction

The styloid process (SP) is a bony projection originating from the petrous part of the temporal bone, extending forward, downward, and slightly inward. This anatomical structure is the core of the styloid apparatus, which functions as a unit with three attached muscles (stylohyoid, styloglossus, and stylopharyngeus) and two ligaments (stylohyoid and stylomandibular). The styloid apparatus originates from the second branchial arch [1]. The potential for ossification of the stylohyoid ligament seems to be the reason behind the abnormal elongation of the process, but the exact triggering factor remains unknown. In contrast, there is no evidence of clinical findings supporting the presence of calcification in the stylomandibular ligament.

The typical symptoms associated with calcification of the stylohyoid ligament or elongation of the styloid processes arise mainly from structures within the parapharyngeal space. These structures include essential anatomical elements, like nerves (IX, X, sympathetic trunk), blood vessels (internal jugular vein and carotid artery), and adjacent soft tissues [2]. Patients typically report unilateral neck and orofacial pain (stylalgia), a sensation of a foreign body in the throat, which can result in difficulty in swallowing solid foods, and auditory symptoms like tinnitus. Less frequent symptoms, such as visual dysfunction, dysgeusia, hypersalivation, headache, dizziness, syncope, and breathing issues, require careful evaluation and differentiation in the diagnostic process. Many of the manifestations associated with Eagle Syndrome (ES) overlap with the typical clinical features of burning mouth syndrome (BMS) or temporomandibular joint disorders (TMD). This overlap creates a potential risk of misdiagnosis [3].

Considerable variations exist in the scientific literature regarding the prevalence of anomalies in the stylohyoid chain (SC). This is likely a result of the subjective nature of diagnosis, arising from the diversity in radiographic assessment criteria and the variability in symptoms. SC anomalies can exhibit significant variability, with reported rates ranging from as low as 0.01% to as high as 84.4% [4,5,6,7,8]. Among individuals with calcification of the stylohyoid ligament or elongation of the styloid process, less than 4% will eventually experience symptoms [9]. Even though the author was not the first to describe it, this medical condition is widely recognized as Eagle Syndrome [10]. 

The recommended treatment modality remains surgery due to its high success rate and low occurrence of postoperative complications. Pharmacological treatment should be considered solely in surgery-related contraindications associated with patient morbidity or when the patient refuses surgery. Any accompanying neurological deficits should be treated as an emergency, with primary or secondary styloidectomy demonstrating genuine effectiveness [11]. There are two primary surgical modalities available: by intraoral or external approach. This study aims to introduce a quick, efficient, and minimally invasive technique of styloidectomy.

## 2. Materials and Methods

### 2.1. Patients

**Figure 1:**
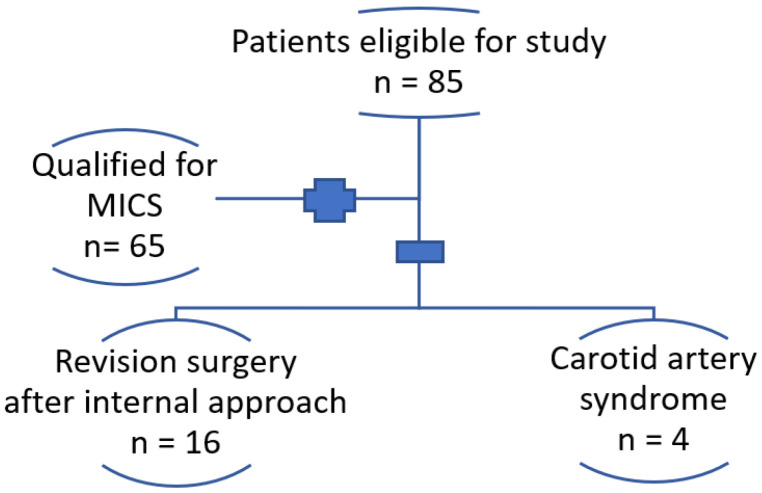
Flowchart of patient selection.

Between September 2021 and March 2023, 85 adult patients (64 females and 21 males aged 16 to 70) underwent styloidectomy procedures performed by the same surgeon at our Institution. Among these patients, 65 had classic stylalgia and underwent minimally invasive cervical styloidectomy (MICS), with nine necessitating bilateral procedures. The patients exhibited symptoms comprising orofacial pain, dysphagia, odynophagia (painful swallowing), sensation of a foreign body in the throat, and headaches. Nine patients were operated on bilaterally. The remaining 20 individuals were not eligible for MICS and underwent traditional, more extensive surgical procedures with broad neck exposure. This was necessitated by secondary scarring following an incomplete resection through the intraoral approach and the requirement for decompression of the internal carotid artery (Figure 1).

All patients underwent a comprehensive medical history review and a thorough head and neck examination. Conditions like temporomandibular joint disorders (TMDs), burning mouth syndrome (BMS), neuralgia, ear nose and throat (ENT) diseases, and cephalalgias were exclusion criteria. Each patient was evaluated on the basis of neck angioCT as a preferred diagnostic tool. This step aimed to confirm the presence of styloid chain anomalies and assess variability in vascular anatomy to plan the surgical treatment.

The patients’ characteristics are presented in Table 1.

### 2.2. Surgery Planning

**Figure 2:**
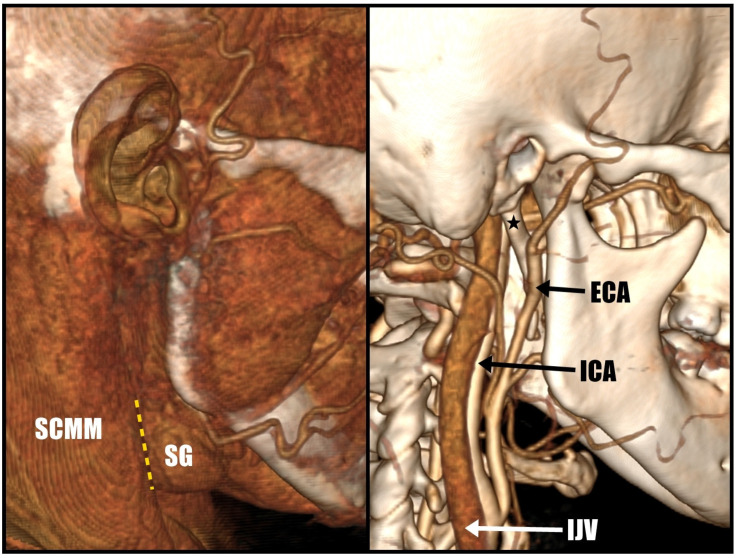
Proper surgical planning on angioCT. The styloid process is marked with “★”. Entry point to SHC is marked with “---“.

Before proceeding with the surgery, conducting a thorough analysis of the angioCT scan is essential to identify crucial anatomical structures. During the procedure, the surgeon may encounter the sternocleidomastoid muscle (SCMM), submandibular gland (SG), and vascular structures such as the external jugular vein (EJV), internal jugular vein (IJV), external carotid artery (ECA), and internal carotid artery (ICA). Any anomalies should be identified before the operation. The entry point to the stylohyoid chain lies between the submandibular gland and the sternocleidomastoid, maintaining a safe distance from the potential region of the marginal branch of the facial nerve (Figure 2).

### 2.3. Surgical Technique

MICS is performed in general anesthesia without a neuromuscular blocking agent. The patient is placed in a supine position with the head extended and turned to the contralateral side. The neck and the lower half of the face are draped in the usual sterile fashion. Intraoperative nerve monitoring is optional yet recommended for inexperienced surgeons. The author recommends the use of surgical loupes and an LED headlamp.

**Figure 3:**
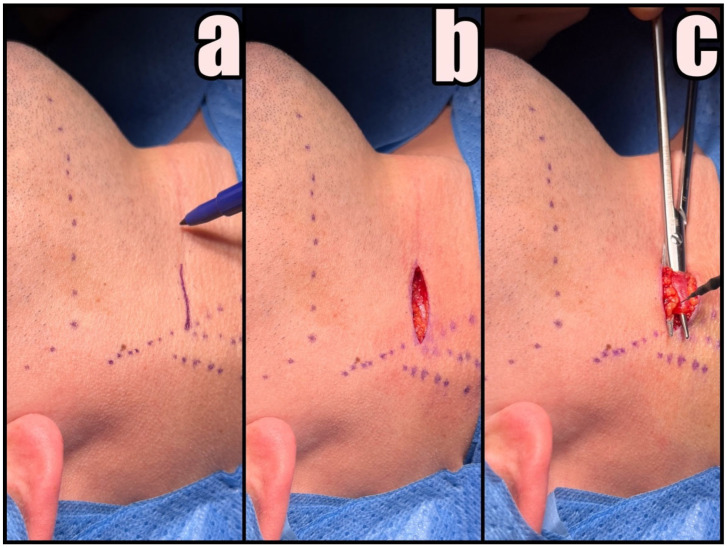
Initial phases of the procedure. (**a**) Skin marking, (**b**) skin and subdermal tissue incision, and (**c**) dividing of platysma followed by blunt dissection.

First, the SCMM and EJV are identified and marked on the skin. A small 3–4 cm skin incision anterior to SCMM is made at least 3 cm below the lower border of the mandible in the preexisting skin crease with no. 15 blade followed by Colorado^®^ microdissection needle to expose the platysma muscle (Figure 3a,b). The marginal branch of the facial nerve and the transverse cervical nerve should be preserved.

Platysma muscle is undermined with dissecting scissors and divided in the middle for subsequent suturing (Figure 3c). If the EJV interferes with the skin incision projection, it should be dissected and shifted backward. There is no need for ligation of any vessel during this procedure. Subsequent stages are performed only by gentle blunt dissection with curved Pean hemostatic forceps and a tupfer gauze ball.

**Figure 4:**
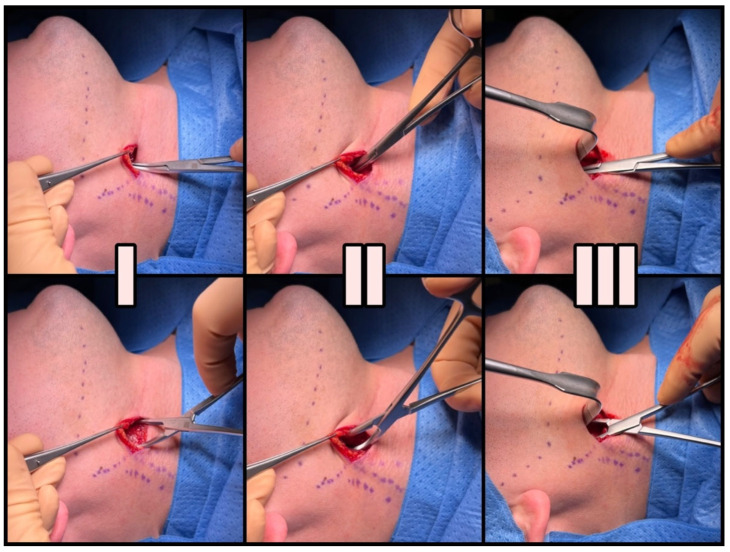
A three-stage technique for accessing the styloid process. (**I**) Undermining the platysma, (**II**) revealing the loose connective tissue, and (**III**) identifying the styloid process.

Blunt dissection maneuvers can be divided into three stages. The initial stage involves releasing the platysma to facilitate more precise identification of the anatomical structures. Following the elevation of the platysma with surgical tweezers, the muscle is bluntly dissected over the area of 1 cm with the gauze pad (Figure 4(I)) Secondly, the forceps are introduced anteriorly to the sternocleidomastoid muscle (SCMM) and posteriorly to the submandibular gland at an angle of 30–45 degrees, aligned in the projection of the mandible angle (gonion point). With few gentle openings loose, cobweb-like connective tissue surrounding deep cervical structures is identified (Figure 4(II)). Blunt dissection ends when the internal jugular vein (IJV) and the stylohyoid muscle (SHM) crossing over the posterior belly of the digastric muscle are identified. At this point, the surgeon can use their index finger to identify various bony landmarks, including the hyoid bone, the transverse process of C2, the angle of the mandible, and the styloid process. Before proceeding to the final phase, it is necessary to insert the Kocher–Langenbeck retractor into the created tunnel, positioning the tool at a 90° angle to the lower border of the mandible. Care should be taken to prevent the hook from sliding along the margin of the mandible, as this action could potentially damage the marginal branch of the facial nerve. The last stage involves identifying the styloid process. The SHM and IJV determine the direction of the blunt preparation, which aligns with the hypothetical line established by the course of the EJV marked earlier. With the forceps curved towards the neck, the stylohyoid ligament or ossified stylohyoid tract (depending on the elongation) is reached after a few gentle movements (Figure 4(III)). With substantial expertise, these steps can be executed using just three moves.

**Figure 5:**
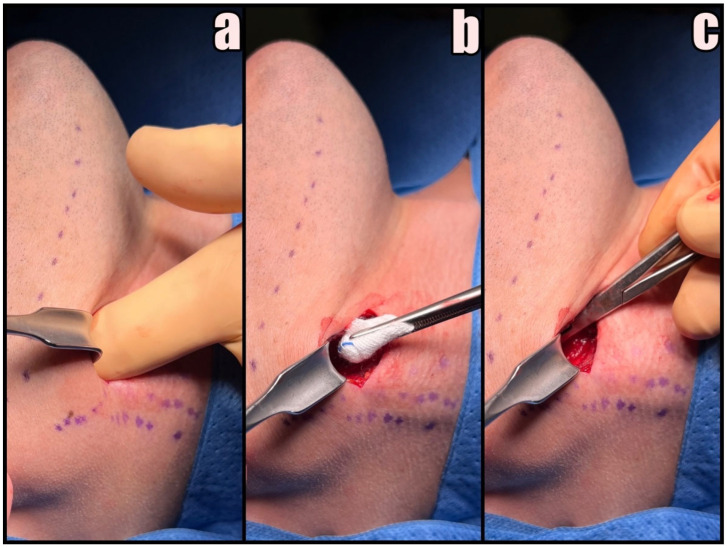
Uncovering the styloid process. (**a**) Using the index finger for verification, (**b**) delicate preparation with a tupfer, and (**c**) final check of the inferior surface of the cranial base.

Once the elongated styloid is identified, with the index finger serving as a guide, the retractor’s position is adjusted along the axis of the process (Figure 5a). At this point, gentle upward traction is applied to the external carotid artery. By utilizing gauze balls for blunt dissection and a Molt elevator against the outer bone surface of the styloid, access to the cranial base is achieved. Verification of this region is confirmed by identifying the sheath enveloping the process, known as the vaginal process of the temporal bone (Figure 5b). The vaginal sheath can be carefully opened using blunt Pean forceps (Figure 5c).

**Figure 6:**
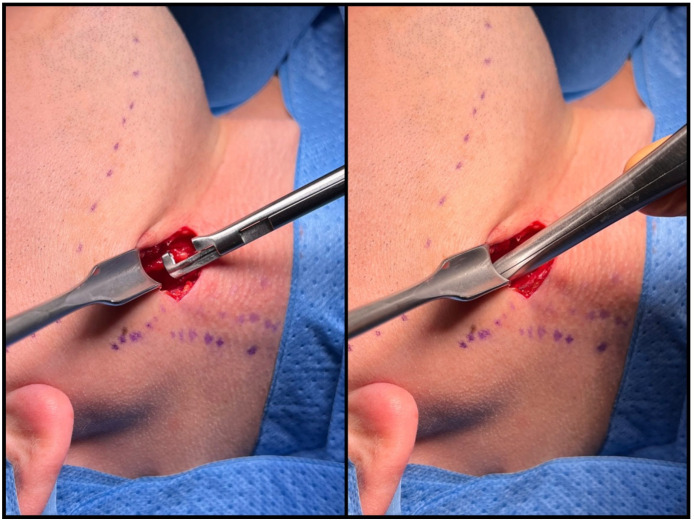
Kerrison forceps inserted with their tips directed medially.

We recommend using Kerrison rongeur to carefully remove the styloid process from its posterior surface, ensuring a 1 cm distance from the base of the skull (Figure 6). It is crucial to orient the forceps’ tips medially during this maneuver to diminish the risk of damaging the facial nerve and potential rupture of the IJV. If the styloid process exceeds thickness of 5 mm, it is advisable to employ forceps from both sides instead of attempting to fracture it. When dealing with a styloid process in the vicinity of big vessels, a diamond drill or piezosurgery should be used to eliminate lateral movements during the cutting process.

**Figure 7:**
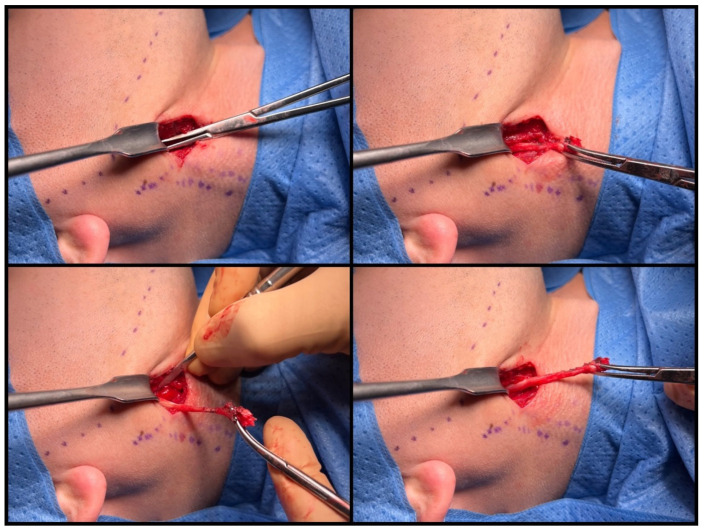
Dissection of the elongated styloid process.

The styloid process is securely held using curved vascular forceps and carefully removed. Muscles are detached with a sharp periosteal elevator assisted by the Colorado^®^ microdissection needle. The styloid process can be safely extracted when solely supported by the stylohyoid ligament, which is subsequently cut (Figure 7).

**Figure 8:**
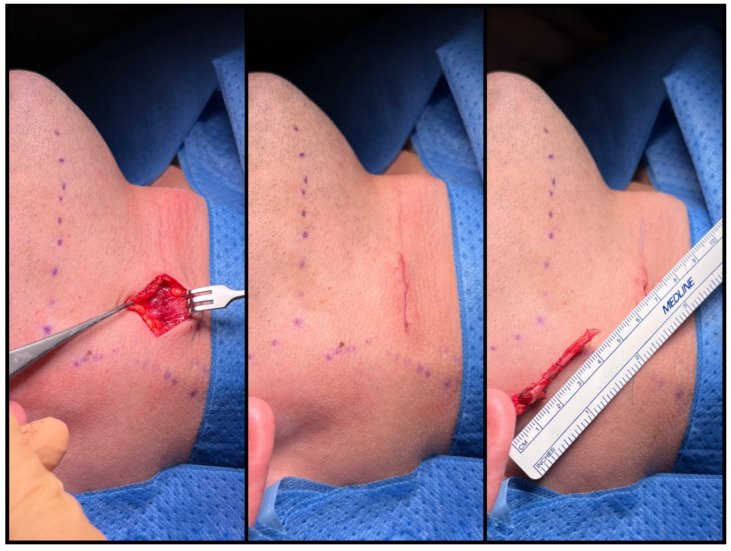
Multi-layered wound closure with intradermal skin sutures.

To ensure that no sharp edges remain, the base of the skull is palpated. Any residual fragments can be easily removed with straight Pean or wire-twisting forceps. The wound is closed in layers, with meticulous suturing of the platysma muscle. Draining is usually unnecessary. Intradermal sutures are used for skin closure (Figure 8).

## 3. Results

We have performed a total of 65 minimally invasive styloidectomies. Our patients ranged in age from 16 to 70, with an average age of 38 and a male-to-female ratio of 2.6 to 1. Complete demographic data are presented in Table 1.

**Figure 9:**
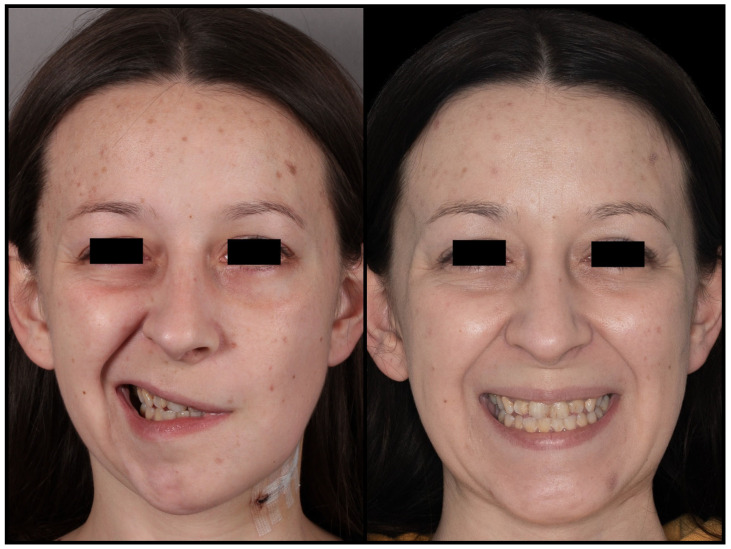
Spontaneous resolution of postoperative facial nerve weakness during follow-up.

The shortest styloid process measured 26 mm, while the longest measured 80 mm. Bilateral ES was observed in nine patients, which accounted for 13.8% of the cases. The recovery proceeded without significant complications, except for a transient issue related to temporary facial nerve weakness caused by retraction (Figure 9).

The average duration of the MICS procedure was 36 min. No instances of bleeding requiring vessel ligation were encountered during the surgeries. The removed styloid fragments ranged in length from 2 cm to 7 cm. We defined treatment success as either complete symptom remission or significant improvement, with a minimum follow-up period of 6 months. This result was achieved in 63 out of 65 patients, representing a 97.0% success rate. Interestingly, the one case where symptoms persisted involved the shortest styloid process. All patients expressed satisfaction with the aesthetic outcome of the scar formation. Detailed results are presented in Table 2.

## 4. Discussion

We have taken the initiative to develop a simplified styloidectomy technique. This approach was designed to reduce the risk of potential complications, shorten the operative time, and facilitate the healing process. Our experience underscores this technique’s importance, effectiveness, and safety, mainly because medical professionals often underestimate the possibility of stylohyoid chain anomalies. Consequently, patients are referred to psychiatrists or forced to look for help on their own.

The symptomatic elongation of the styloid process has a well-documented historical presence.

### 4.1. History

In 1543, Vesalius observed the first anomalies of the stylo-hyoid ligaments in animals [12]. One century later, in 1652, Marchetti described the elongated styloid process for the first time as a paraphysiological yet non-harmful condition [13]. By the end of the 19th century, Stirling wrote about peculiar bony structures of unknown origin that could be palpated in the throat [14]. In 1907, Dwight established a groundbreaking link between orofacial pain and variations in the anatomy of the stylohyoid ligament, providing one of the earliest insights into the condition’s pathogenesis [15]. In 1927, Garel independently analyzed the symptomatology; in 1932, Bernfeld introduced the first successful surgical treatment of the disease [16,17]. Notably, references to the Garel–Bernfeld syndrome can be found in various publications from the past [18,19]. Between 1937 and 1962, Eagle researched this condition, presenting publications that described the symptomatology and proposed surgical treatment [20,21,22]. Eagle has identified two distinct forms: the classic form associated with tonsillectomy and the carotid artery syndrome. The classic form was supposed to be a complication when scar tissue forms towards the elongated styloid process, leading to facial, glossopharyngeal, and vagus nerve irritation. The carotid artery syndrome is caused by the mechanical compression of the long styloid process on the sympathetic fibers of the carotid plexus. Currently, there is no recognized association between tonsillectomy and the occurrence of symptoms, and carotid artery syndrome has more complex symptomatology. Frequently, more descriptive terms are used, such as stylalgia, styloid process neuralgia, or previously stressed stylohyoid chain anomalies [23].

### 4.2. Symptoms

Individuals afflicted with ES frequently present with a broad spectrum of nonspecific issues spanning various medical specialties. Often, the diagnosis takes many years. Predominant symptoms include unilateral orofacial and neck pain, the sensation of a foreign body in the throat, and auditory symptoms such as ear pain or tinnitus, often provoked during chewing, speaking, yawning, or head movements. Other documented symptoms include a range of neurological and vascular issues. Neurological symptoms include dizziness, one-sided headaches, visual disturbances, and excessive salivation (sialorrhea). At the same time, vascular complications encompass transient ischemic attacks (TIAs), Horner’s syndrome (marked by miosis, ptosis, and anhidrosis), and, in some cases, ischemic strokes due to internal carotid stenosis [24,25].

Pain serves as the predominant symptom driving patients to seek a diagnosis. This pain is often characterized by its extreme intensity, frequently described as sharp or shooting, primarily localized in the pharynx and neck. Nevertheless, it can also manifest in various other regions, including the floor of the mouth, jaws, temporomandibular joint, ear, face, orbit, and skull base. Regrettably, numerous other medical conditions exhibit similar symptomatology. Therefore, arriving at a definitive diagnosis necessitates a comprehensive evaluation and a meticulous differential diagnosis. A proper diagnosis should primarily encompass the differential diagnosis of dental, maxillofacial, ENT, neurological, or ophthalmic issues.

### 4.3. Diagnostic Imaging

**Figure 10:**
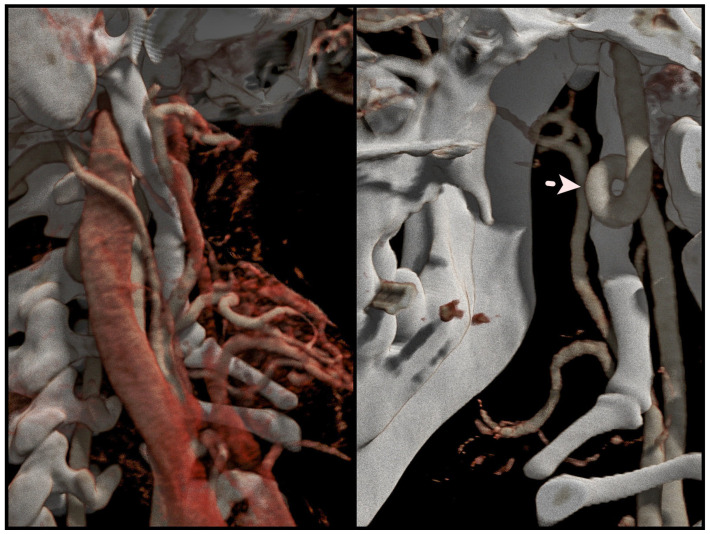
A high-quality three-dimensional reconstruction of the vascularity in the stylohyoid region, revealing kinking of ICA as indicated by the arrow (own material).

Diagnostic imaging is crucial for identifying styloid chain anomalies, and various techniques can be employed for this purpose, including panoramic radiographs, cone-beam computed tomography (CBCT), and computed tomography (CT). Magnetic resonance imaging (MRI) may be considered to visualize soft tissues for a more comprehensive assessment. Among these imaging modalities, CT is the preferred choice due to its three-dimensional reconstruction capabilities, which allow precise measurements of the process’s length, tip deviation, and proximity to adjacent anatomical structures [26]. The authors recommend their protocol for diagnostic imaging. CBCT is used for swift screening assessment, offering high accuracy with the potential for a lower radiation dose. Upon diagnosing ES, neck angioCT is performed to analyze the anatomy of blood vessels, thus facilitating meticulous surgical planning. This becomes especially crucial when anatomical variations are present (Figure 10).

**Figure 11:**
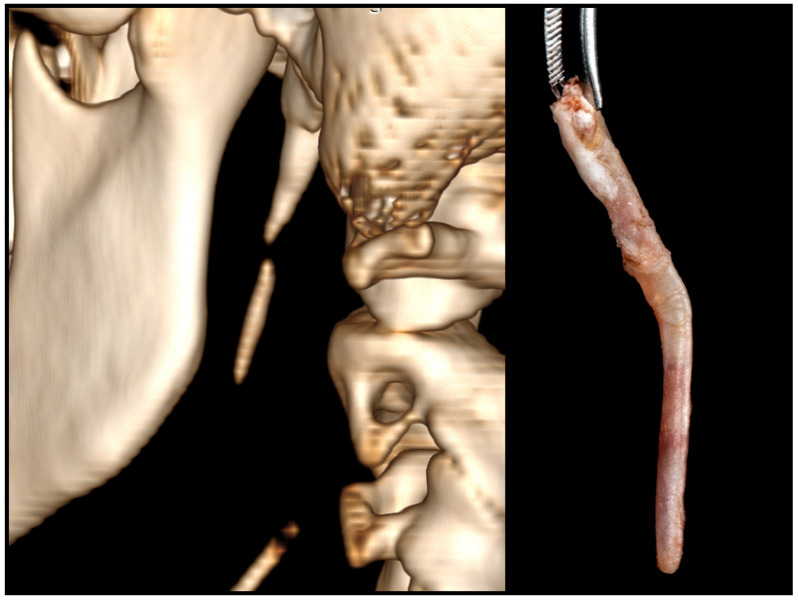
Bony fragments of the stylohyoid chain, individually observed on the 3D CT scan, appeared structurally connected by a calcified ligament upon removal.

Obtaining correct measurements is a critical factor for diagnosis. The physiological length of the styloid process varies, with the average size not exceeding 3 cm [27]. The quickest and most accurate approach involves a 3D reconstruction of the DICOM files. It is essential to ensure that the longitudinal axis of the stylohyoid chain aligns parallel to the measurement plane. In cases where there is angulation or separation of the bony segment, each fragment and the gaps between them should be measured individually. Any vacant spaces detected correspond to the abnormal stylohyoid ligament (Figure 11). The actual length of the elongated styloid process is the sum of all these measurements.

### 4.4. Treatment Options

Conservative treatment can address Eagle Syndrome, but surgical resection is the treatment of choice [28]. Conservative management of stylalgia may be suitable for patients without neurological deficits, especially when addressing less severe pain or when surgery is contraindicated. This approach entails the use of manual therapy, non-steroidal anti-inflammatory drugs, long-acting analgesics, corticosteroids, antiepileptics, or muscle relaxants, along with the possibility of administering injections of anesthetics or steroidal anti-inflammatory medications into the tonsillar fossa [29,30,31]. Clinical research has shown that consistent long-term relief is not achieved with conservative treatment, and symptoms tend to recur within 6 to 12 months after such treatment [32,33]. Lidocaine injection might be a valuable tool for evaluating the potential effectiveness of styloidectomy procedures. A 1 mL dose of 2% lidocaine is carefully administered into the anterior pillar and approximately 1–2 cm into the tonsillar fossa. If the patient experiences temporary relief of symptoms shortly afterward, the test is considered positive, affirming Eagle Syndrome’s diagnosis [34]. Surgery might be performed by intraoral or extraoral approach.

The intraoral approach can be executed with endoscopy or robotic surgery [35,36]. Depending on the angulation of the styloid and transoral accessibility assessed by palpation, it can be managed with or without tonsillectomy. Its primary notable advantage is the lack of skin scars. However, the transoral route has certain drawbacks, including the exposure of parapharyngeal spaces to the intraoral environment, the potential risk of nerve damage, severe bleeding that may necessitate a secondary cervical approach, and the necessity of following a liquid diet during the postoperative period [37]. The higher risk of infection necessitates the routine administration of perioperative antibiotics. Additionally, specific indications are associated with the appropriate length and positioning of the styloid process within the parapharyngeal space [38]. Studies indicate good results with an intraoral surgical approach to the styloid process [39,40]. However, it is important to note that most of these studies consist of case reports or case series [41,42,43]. Our research underscores the potential concern of a moderate recurrence rate of symptoms linked to incomplete removal of the elongated styloid process through the transoral approach. Notably, 18.82% of our patients had previously undergone this procedure.

External styloidectomy can be performed using a retroauricular, preauricular, perimandibular, or cervical approach. The risks associated with each method vary depending on the incision’s location. The primary advantages of the cervical approach include maintaining an aseptic surgical field with a minimal risk of surgical site infection with adequate exposure of the styloid process and adjacent structures. The only disadvantage associated with it is the potential risk of damaging the marginal branch of the facial nerve [44]. However, it can be minimized by using a nerve stimulator and correct dissection plane. The duration of the surgery and aesthetic outcome rely on the surgeon’s expertise and should not be considered a downside of the technique. Advantages include broad access with excellent visibility of the skull base area and major vascular trunks and the feasibility of any stylohyoid chain anomaly treatment. The use of prophylactic antibiotic therapy is not necessary.

Based on our experience, we recommend a small cervical approach within a preexisting skin crease located 3 cm below the lower border of the mandible, anterior to the sternocleidomastoid muscle. It should be noted that posterior-oriented incisions risk damage to greater auricular nerve. In contrast, anterior and more superior approaches pose a risk to the submandibular gland and consequent marginal branch of facial nerve damage. Awareness is essential to identify and protect the transverse cervical nerve within this anatomical region. We do not recommend the intraoral approach due to its limitations and potential vascular and inflammatory complications risk. The advantage of an invisible scar should not influence the choice of a safer and more reliable technique. Minimally invasive styloidectomy is a safe and well-tolerated technique for ES treatment for experienced head and neck surgeons.

## 5. Conclusions

Minimally invasive styloidectomy proves to be an effective option for addressing the stylohyoid chain anomalies. This approach presents numerous compelling advantages, including outstanding success rates, minimal risk of complications, and favorable aesthetic outcomes, all achieved within a short duration of time. These benefits indicate a substantial potential for reducing recovery time and the risk of symptom recurrence.

As a result, there is a growing likelihood that minimally invasive cervical styloidectomy will become the preferred option for most Eagle Syndrome patients, surpassing traditional extensive neck approaches or intraoral procedures. Nonetheless, it remains vital to underscore the essential role of proper diagnosis through differential diagnosis and meticulous surgical planning based on neck angioCT.

## Figures and Tables

**Table 1:** Patients’ characteristics.

PATIENT (*n* = 65)	
AGE IN YEARS (MEAN)	16–70 (38)
GENDER, *N* (%)	
male	18 (27.7%)
female	47 (72.3%)
SITE, *N* (%)	
right	28 (43.1%)
left	37 (56.9%)
BILATERAL STYLALGIA, *N* (%)	9 (13.8%)
LENGTH OF STYLOID (MEAN)	2.6–8 cm (42.5 cm)

**Table 2:** Outcomes and Complications.

PATIENTS (*n* = 65)	
OPERATIVE TIME IN MINUTES (MEAN)	15–65 (36)
LENGTH OF REMOVED STYLOID IN CM (MEAN)	2–7 (43)
COMPLICATIONS	No. of patients (%)
Ipsilateral temporary facial nerve weakness	1 (1.5%)
OUTCOMES	No. of patients (%)
Complete remission of symptoms	56 (86.2%)
Significant improvement with partial remission of symptoms	7 (10.8%)
Partial relapse without the need for surgical treatment	1 (1.5%)
No improvement	1 (1.5%)
OVERALL SUCCESS RATE	63 (97.0%)
SATISFACTION WITH THE AESTHETIC RESULT	65 (100%)

## Data Availability

Restrictions apply to the availability of these data. Data were obtained from patients treated at the Department of Cranio-Maxillofacial Surgery, Cracow, Poland, and cannot be shared, in accordance with the General Data Protection Regulation (EU) 2016/679.
